# Effects of Freeze-Thaw Cycles on Bioaccessibilities of Polycyclic Aromatic Hydrocarbons

**DOI:** 10.3390/toxics12060413

**Published:** 2024-06-05

**Authors:** Hui Dong, Ze Wu

**Affiliations:** 1 School of Karst Science, Guizhou Normal University, Guiyang 550025, China; 201808010@gznu.edu.cn; 2 College of Eco-Environmental Engineering, Guizhou Minzu University, Guiyang 550025, China

**Keywords:** PAHs, freeze-thaw cycles, aging, bioaccessibility, desorption kinetics

## Abstract

The bioaccessibility of particle-bound hydrophobic organic contaminants, such as polycyclic aromatic hydrocarbons (PAHs), and the factors influencing their re-release are crucial for assessing potential human health risks via inhalation and hand-mouth exposure. However, the mechanisms by which various factors affect the re-release of PAHs in body fluids, particularly in response to environmental changes like freeze-thaw cycles, remain unclear. To obtain a better understanding, an in vitro method was employed to investigate the re-release processes of PAHs from different soil types (ferrallitic soil and calcareous soil) in simulated body fluids (simulated lung fluid and simulated saliva) under varying freeze-thaw conditions (0, 15, and 30 cycles). The findings indicated that the bioaccessibilities of phenanthrene and pyrene decreased with the frequency of freeze-thaw cycles, which were constrained by soil nature and simulated body fluids compositions as well. Additionally, this study observed that the portion of reversible adsorption of PAHs declined after exposure to freeze-thaw cycles in a nonlinear manner, suggesting that the potential human health risk associated with PAHs could be mitigated due to the “aging effect” which occurred as PAHs became less bioaccessible over time. These results underscore the importance of considering the characteristics of pollutants, body fluids, and environmental media when conducting a precise assessment of the human health risks posed by such contaminants.

## 1. Introduction

Polycyclic aromatic hydrocarbons (PAHs) are pervasive, persistent organic pollutants, predominantly originating from the incomplete combustion of fossil fuels and biomass [1]. Recognized for their teratogenic, carcinogenic, and mutagenic properties, 16 types of PAHs have been prioritized as pollutants by the United States Environmental Protection Agency (USEPA), underscoring their significant threat to human health and attracting considerable attention [2]. Due to their hydrophobic nature, PAHs tend to adsorb onto organic matter–rich environmental media, such as soil and sediment, upon release into the environment. These pollutants can be re-released when environmental conditions fluctuate, posing ongoing risks to both the ecological environment and human health. Hence, a thorough understanding of the re-release dynamics of PAHs from environmental media is essential for accurately assessing the associated environmental and health risks.

Human exposure to PAHs occurs through various pathways, including ingestion and inhalation, with the latter two being the most prevalent [3]. Children, in particular, are at an increased risk of absorbing environmental pollutants from soils and dust due to their behavioral patterns, such as hand–to–mouth activity [4]. In vitro assays have been instrumental in determining the portion of pollutants that desorb from particles upon contact with biological fluids [5]. Extensive research has demonstrated that considering the total pollutant load in media overestimates health risks. For instance, Zhang et al. [6] employed various digestion models to extract PAHs from contaminated soil, revealing bioaccessibility rates below 13.4% for individual PAHs. In soils treated with contaminated wastewater, the bioaccessibility of PAHs in the small intestine was higher, peaking at 53% [7]. Similarly, the bioaccessibility of PAHs in PM2.5 in the respiratory tract reached a maximum of 24.5% [8]. Xie et al. [9] utilized Modified Gamble’s Solution (MGS) and Artificial Lysosomal Fluid (ALF) to extract PAHs from e-waste particles, with results indicating the highest release rate nearing 90% and consistently higher bioaccessibility in ALF compared to MGS. Furthermore, the “aging effect” refers to the decline in extractability and bioavailability of soil-bound PAHs over time, reducing their availability to microorganisms and other ecological receptors [10,11]. Wei et al. [10] observed a significant decrease in the available extraction rate of pyrene with increasing contact time, particularly in the rapid desorption fraction. After 150 days of exposure, the desorption rate of phenanthrene dropped from 82% to 65%, with pyrene showing a less pronounced change. This aging effect leads to an increase in the irreversible fraction of PAHs, thereby diminishing ecological risks [12].

In summary, the re-release of PAHs is influenced by multiple factors, including the number of rings, hydrophobicity, the nature of the media, the properties of the extractant, and the placement in the human body. Current research predominantly focuses on the bioaccessibility of adsorbed PAHs in the digestive and respiratory tracts, with less emphasis on the processes and mechanisms governing these dynamics. Given the significance of inhalation and hand-to-mouth exposure pathways for particle-bound PAHs, further investigation into these processes and mechanisms is warranted. Additionally, environmental conditions are subject to change, which can alter the physical and chemical properties of soil and other media, subsequently affecting the re-release of adsorbed PAHs. For example, freeze-thaw cycles can lead to the rearrangement of soil particles and alterations in soil structure, impacting the re-release processes of adsorbed PAHs. The implications of these changes on the re-release of PAHs and their potential impact on human health risks require further exploration.

Unraveling the re-releasing process of particle-bound PAH in body fluid may provide insight into its fate and related drivers, allowing for a more precise estimation of the potential human health risk of PAH. The objectives of the present study were (i) to compare the discrepancies in bioaccessibilities of selected PAHs before and after freeze-thaw cycles, (ii) to employ the desorption kinetics model to reveal the re-release process of PAHs in simulated body fluids, and (iii) to identify the key drivers of responding to environmental change in the bioaccessible processes. This study hopes to enhance understanding of the geochemical dynamics of PAHs and provide critical information for risk assessors in accurately characterizing and prioritizing contaminated sites.

## 2. Materials and Methods

### 2.1. Sorbents Preparation

For this study, two distinct types of surface soils were utilized as geosorbents: ferrallitic soil (FS); and calcareous soil (CS). The ferrallitic soil was obtained from the Maolan National Reserve in Libo, while the calcareous soil was sourced from the Duxi Forest Park in Guiyang, both located in Guizhou Province, Southwest China. The soil samples were carefully collected from the upper 0 to 20 cm layer and air-dried to remove moisture. Subsequently, the soil was sieved through a 4 mm stainless-steel mesh to eliminate any roots and stones, ensuring a uniform particle size.

The sieved soil was then meticulously ground using a mortar and pestle to further homogenize the sample. Finally, the soil was passed through a 150 μm stainless-steel sieve for the bioaccessibility experiments. Although these soil particles might not have perfectly replicated the characteristics of particles that are actually inhaled or ingested, they served as suitable proxies for in vitro assays. These assays were designed to reveal the interactions between soil particles and PAHs in simulated body fluids, allowing us to evaluate the effects of various assay parameters on the behavior of these particles at the interface.

### 2.2. Simulated Body Fluids Preparation

The background solution used in this study contained 0.005 M CaCl_2_ and 0.001 M NaHCO_3_. Two artificial body fluids, Gamble’s solution (namely, simulated lung fluid) and simulated saliva, were employed. The detailed compositions of these simulated body fluids are presented in Table 1. To minimize the risk of precipitation, each component was added in the order presented. All solutions were prepared with Milli-Q water, and sodium azide (NaN_3_) with a concentration of 100 mg L^−1^ was added to prevent the degradation of PAHs by microorganisms. Organic reagents (guaranteed reagents) were purchased from Sigma-Aldrich Chemical Co. (Shanghai, China).

### 2.3. Soil Spiking and Aging

**Figure 1:**
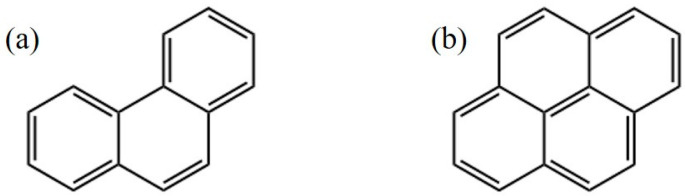
Structural formulas of (**a**) phenanthrene and (**b**) pyrene.

Phenanthrene (C_14_H_10_) and pyrene (C_16_H_10_), two common probe PAHs, were selected as target compounds (Figure 1). These PAHs were initially dissolved in methanol (HPLC grade, Merck, Germany) to create stock solutions with concentrations of 1000 μg L^−1^ for phenanthrene and 100 μg L^−1^ for pyrene. Subsequently, soil particles were spiked with the target PAHs to achieve concentrations of approximately 20 mg kg^−1^ for phenanthrene and 8 mg kg^−1^ for pyrene at a temperature maintained at 21 ± 1 °C for the bioaccessibility kinetics experiments. Prior to incubation, the concentrations of spiked soil PAHs were confirmed analytically. The determination of solid-phase PAH concentrations was performed in accordance with the method established by Huang and Pignatello [15]. Briefly, approximately 1 g of soil was placed into a 20 mL glass vial, followed by the addition of a methanol-water solution in a 4:1 volume ratio, leaving approximately 1 mL of headspace within the vial. The vials were then sealed and shaken horizontally in a water bath at a temperature of 85 °C for 8 h. Post-extraction, the vials were centrifuged at 3000× *g* for 30 min, after which, the supernatant was carefully collected for further analysis.

The soil samples spiked with PAHs were transferred into 50 mL Teflon centrifugation tubes and maintained under moist conditions. They were then subjected to a freeze-thaw process by being frozen at −18 ± 1 °C for 24 h and subsequently thawed at 21 ± 1 °C in a temperature-controlled chamber in the dark for 24 h. This cycle was repeated 15 times (FT15) and 30 times (FT30) for the respective samples to simulate the process of repeated freezing and melting of the surface soil with varying frequency in the natural environment within a short time. Following the freeze-thaw cycles, the samples were freeze-dried under vacuum conditions for subsequent analysis.

### 2.4. Desorption Kinetics Experiments

The desorption kinetics experiments were conducted in duplicate. Briefly, 20 mg of PAH-spiked soil sample was placed into a glass vial with Teflon-lined caps. Subsequently, 15 mL of simulated body fluid was added to the vial, ensuring that the headspace was minimized to less than 0.1 mL. The vials were then securely sealed and horizontally placed into a temperature-controlled shaker to incubate at 37 ± 1 °C with a shaking speed of 120 rpm. The incubation periods were systematically varied as follows: 15 min; 30 min; 1 h; 2 h; 8 h; 24 h; 48 h; 1 week; and 2 weeks, with all incubations conducted in the dark. At each predetermined time interval, the vials were centrifuged at 3000 rpm for 20 min. Following centrifugation, approximately 2 mL of the supernatant was sampled and transferred into a 5 mL glass vial containing pre-weighted methanol (approximately 2 mL) to stabilize phenanthrene and pyrene within the vial. These vials were then stored at 4 °C in a dark environment to await subsequent analytical measurements. A control experiment of no sorbent in the vial was conducted simultaneously to quantify the loss of solute during experiments and was used as a reference for data reduction.

### 2.5. Sample Analysis and Data Calculation

#### 2.5.1. Sample Analysis

The concentrations of phenanthrene and pyrene were determined using ultra-high-performance-liquid-chromatography (1290 UHPLC, Agilent, Santa Clara, CA, USA) equipped with a diode array UV detector (DAD) (G4212A), a fluorescence detector (FLD) (G1321B), and an auto-sampler unit (G4226A), employing an Eclipse XDB-C18 column (2.1 × 150 mm, 3.5 μm) at a column temperature of 37 °C. Phenanthrene and pyrene were analyzed using DAD at wavelengths of 254 nm and 272 nm, respectively, and using FLD with excitation/emission wavelengths of 254 nm/366 nm and 270 nm/392 nm, respectively.

The sorbate concentration in the supernatant or initial solution was calculated by adjusting the UHPLC-measured sorbate concentration with a corresponding dilution factor based on the mass ratio of methanol to water in the mixture.

The total organic carbon (TOC) of each sample was determined using the dry combustion method with an elemental analyzer (Vario El III, Langenselbold, Germany). Prior to analysis, the soil sample underwent treatment with 0.5 mol L^−1^ HCl (with a volume ratio of soil to HCl solution of approximately 1:30) to remove inorganic carbon. Soil particle-size distributions were determined following the hydrometer method (ASTM D422-63). Two blind replicates were included in the analysis, and good repeatability was obtained (<3%).

#### 2.5.2. Data Calculation

Desorption kinetics from soils or sediments are often characterized by fast processes followed by a plateau of slower progress [16]:

(1) $$S_{t}/S_{0}=F_{rap}e^{{-k}_{rap}t}+F_{slow}e^{{-k}_{slow}t}$$

where S_t_ (mg kg^−1^) is the PAH content in the soil at time t (h) and S_0_ (mg kg^−1^) at the start of the experiment; F_rap_ and F_slow_ (–) are the rapidly and slowly desorbing fractions; k_rap_ and k_slow_ (h^−1^) are the rate constants of rapid and slow desorption compartments, assuming that k_slow_ ≪ k_rap_. We additionally assumed that the two defined fractions covered the

(2) $$F_{rap}+F_{slow}=1$$

Parameters in Equations (1) and (2) were determined by direct curve fitting, minimizing the cumulative squared residuals between experimental and calculated values of S_t_/S_0_ (Origin 2022).

The bioaccessibility (%BA) of PAH was calculated by adding the percentages released in simulated lung fluid and simulated saliva:

(3) $$\%BA=\frac{BA\;extracted\;PAH}{total\;PAH}\times 100\%$$

The Mann-Whitney U test was used to verify the significance of variations between compounds, soils, and simulated body fluids, while the Jonckheere-Terpstra test was used to determine the significances of variations among groups of freeze-thaw cycles. All statistical analyses were performed using SPSS (v23.0, IBM Corp, Armonk, NY, USA). All the figures were prepared using GraphPad Prism 9.

### 2.6. Quality Assurance and Quality Control

A method blank, a spiked blank, and a pair of matrix-spiked sample duplicates were processed and analyzed in parallel. A total of 20% of the samples were selected to calculate the recovery, which was (87 ± 12)% for phenanthrene and (95 ± 11)% for pyrene.

## 3. Results and Discussion

### 3.1. General Shape

**Figure 2:**
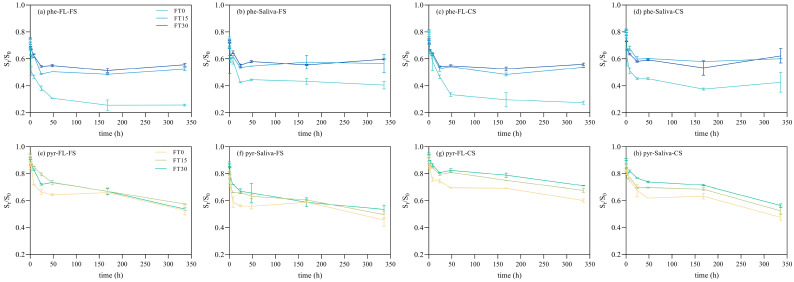
Desorption kinetics curves phenanthrene (**a**–**d**) and pyrene (**e**–**h**) in different scenarios (Note: F_rap_—rapid desorbing fraction; phe—phenanthrene; pyr—pyrene; FS—ferrallitic soil; CS—calcareous soil; FL—simulated lung fluid; FT—freeze-thaw cycle).

The desorption kinetics of phenanthrene and pyrene exhibited a typical biphasic pattern, as illustrated in Figure 2. Initially, within the first 0 to 24 h, a rapid desorption phase occurred, releasing approximately 60% to 90% of the total desorbed amount. Subsequently, this initial rapid phase transitioned into a second phase characterized by significantly slower desorption rates, maintaining an apparent balance until the end of the experiment. Notably, certain samples subjected to treatment reached the transition point between the two phases more swiftly compared to the untreated FT0 group. As presented in Table 2 and Table 3, the k_rap_ values varied from 0.37 h^−1^ to 6.90 h^−1^, which was consistent with the findings of Cornelissen et al. [17]. It reported that PAHs desorbing from polluted sediments typically exhibited rapid desorption rate constants above 0.1 h^−1^. The slow desorption rate constants (k_slow_) were significantly lower than the k_rap_ values, which supported the assumption of the bi-phasic desorption model. However, no clear correlations were found between k_slow_, the PAH individuals, and the soil types.

These observations align with the findings of Barnier et al. [15], who reported that PAHs desorbing from aged industrial soils exhibited a relatively swift desorption step within the first 10 to 15 h, accounting for 40–60% of the total desorbed amount. The observed desorption kinetics pattern in this research aligns with those reported in previous studies on e-waste [9], contaminated soils [6], and natural organic matter [18]. Nonetheless, discrepancies among these studies, particularly in transit time and the percentage of the rapid desorbed fraction, likely stem from differences in the sorbent used, the duration of aging, and the extractant properties.

### 3.2. Contribution of Soil Nature

**Figure 3:**
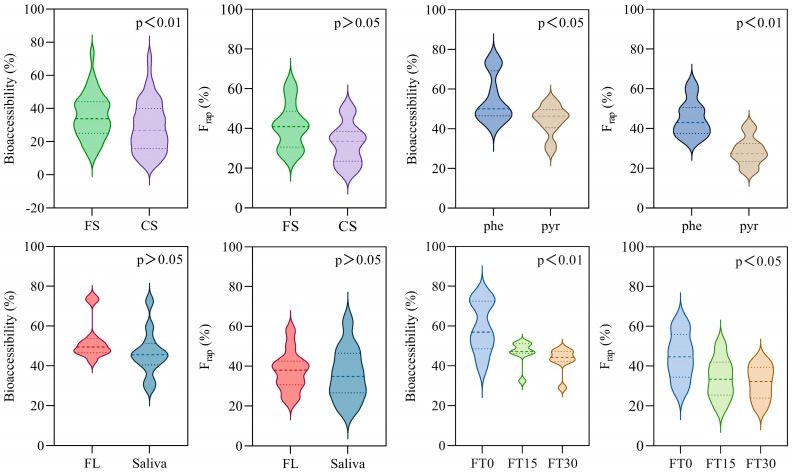
Variations in bioaccessibilities and rapid desorbing fractions of PAHs in different scenarios (Note: abbreviations are similar to Figure 2).

Soil characteristics emerged as a critical factor influencing the bioaccessibilities of PAHs. Calcareous soil (CS), characterized by elevated levels of humin and total organic carbon (TOC) as outlined in Table 4, provided more energetically favorable adsorption sites, thereby exerting a stronger affinity for PAHs. Consequently, the maximum release rates of PAHs from ferrallitic soil (FS) varied from 43.66% to 74.46%, in contrast to the 28.99% to 42.03% range observed in CS. This discrepancy between FS and CS was statistically significant (*p* < 0.01) (Figure 3). Additionally, FS exhibited rapid desorbing fraction (F_rap_) values ranging from 26.43% to 62.29%, while CS showed values between 18.50% and 50.24% (Table 2 and Table 3). The presence of fewer high-energy adsorption sites in FS was associated with greater adsorption reversibility, potentially explaining the higher F_rap_ values observed, although the difference did not reach statistical significance (*p* = 0.068).

Drawing from the hypothesis of Weber and Huang [20], soil organic matter (SOM) can be conceptualized into three distinct domains: the inorganic mineral surface (Domain I); “soft carbon” (Domain II); and “hard carbon” (Domain III). Sorption and desorption of hydrophobic organic compounds (HOCs), such as PAHs, on Domain I are predominantly controlled by partition, characterized by rapid and reversible processes primarily influenced by clay minerals. In contrast, Domains II and III represent two physically and chemically distinct types of carbon: “soft carbon”, which includes humic and fulvic acids; and “hard carbon”, comprising humin, kerogen, and black carbons [21,22]. “Soft carbon” possesses a rubbery or amorphous structure, leading to linear and reversible sorption and desorption of HOCs, while “hard carbon” is more condensed or glassy, leading to slow, nonlinear, and irreversible sorption and desorption processes [20,23].

Furthermore, the elevated “hard carbon” content in CS suggests that the pores within the organic carbon domain are more stable and resistant to deformation caused by freeze-thaw cycles. CS, with its lower C/N and H/C ratios, indicates a more advanced state of condensation and humification in SOM, which confers a stronger affinity and binding capacity for HOCs. Consequently, after several freeze-thaw cycles, the release rates and rapid desorption fractions of phenanthrene and pyrene in CS exhibited significantly less variation than those in FS.

### 3.3. Role of Hydrophobicity

**Figure 4:**
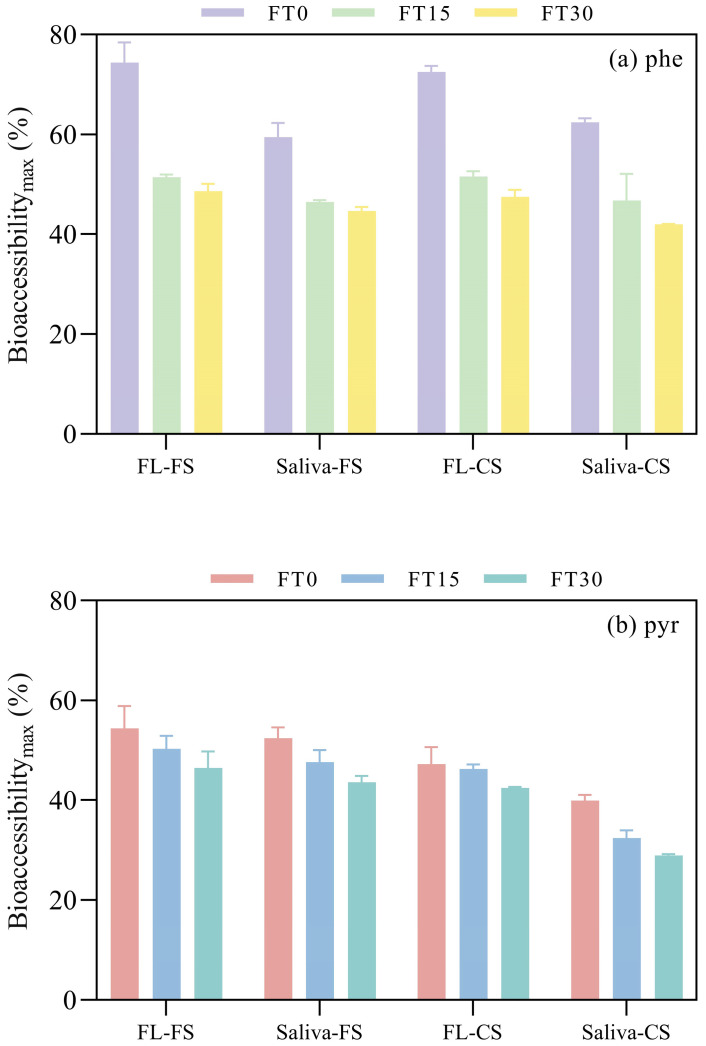
Maximum bioaccessibilities of (**a**) phenanthrene and (**b**) pyrene in different scenarios (abbreviations are similar to Figure 2).

The bioaccessibilities of PAHs varied significantly (*p* < 0.05), as illustrated in Figure 3. Notably, pyrene, with its higher molecular weight and increased hydrophobicity, consistently demonstrated lower bioaccessibility across different scenarios compared to phenanthrene, as depicted in Figure 4. The maximum release rates for phenanthrene spanned from 42.03% to 74.46% (Table 5), surpassing those of pyrene, which ranged from 28.99% to 54.37% (Table 6). The F_rap_ values of phenanthrene and pyrene displayed significant differences (*p* < 0.01), as depicted in Figure 2. Phenanthrene exhibited a more rapid desorption, with fractions ranging from 34.00% to 62.29% (Table 2), surpassing those of pyrene, which ranged from 18.50% to 41.58% (Table 3) across all scenarios. The elevated F_rap_ and k_rap_ values for phenanthrene suggest a potentially higher environmental health risk.

The observed trends in bioaccessibility between lower-ring versus higher-ring PAHs align with expectations, as the transference of compounds from particles to body fluid is predominantly governed by hydrophobicity [9,24,25]. Lower-ring PAHs, being more hydrophilic, tend to exhibit greater bioaccessibility than higher-ring, more hydrophobic PAHs. The discrepancy in release processes between the two PAHs in simulated body fluids is consistent with the fact that pyrene, being more hydrophobic with a higher octanol-water partition coefficient (with the logK_ow_ value of 5.18), tends to remain associated with soil particles in the aqueous environment more than phenanthrene, which has the lower logK_ow_ value of 4.57. Previous research [18] has indicated that pyrene exhibits a higher logK_F_ value across various scenarios, suggesting a stronger affinity to soil particles. The increased number of benzene rings in pyrene results in a larger molecular volume and enhanced hydrophobicity, promoting its distribution and diffusion into soil particles to reduce the entropy of the solution system. Consequently, pyrene demonstrates a stronger adsorption capacity, as indicated by its logK_F_ value, and is more likely to diffuse into the rigid domains of soil organic matter, where it can bind with high-energy adsorption sites, thereby reducing its opportunity to transit into the liquid phase. Furthermore, the properties of pyrene confer a higher hysteresis in soil particles [21], which is associated with a more pronounced decrease in bioaccessibility following exposure to freeze-thaw cycles. According to the calculation by Wang and Brusseau [26], the molecular diameter of phenanthrene is 0.58 to 0.78 nm, while that of pyrene is 0.71 to 0.89 nm. This dimensional discrepancy suggests that pyrene is more susceptible to being sequestered in micropores, which are deformed by freeze-thaw cycles.

### 3.4. Effect of Simulated Body Fluids

The type of simulated body fluid also had an impact on the release rates of PAHs. Higher bioaccessibility values were observed in simulated lung fluid, ranging from 42.48% to 74.46%, which were higher than those in simulated saliva (28.99–62.41%). Unexpectedly, the difference in release rates between the two body fluids did not reach statistical significance (*p* > 0.05). Furthermore, PAHs demonstrated higher rapid desorbing fractions in simulated lung fluid (for phenanthrene: 37.06–57.90%, and for pyrene: 23.00–41.58%) than in simulated saliva (for phenanthrene: 34.00–62.29%, and for pyrene: 18.50–39.00%); yet, this difference was not statistically significant (*p* > 0.05) (Figure 3).

PAHs exhibited distinct release behaviors in the two types of simulated body fluids. Across all experimental conditions, the maximum release rates were generally higher in simulated lung fluid compared to simulated saliva, which could be attributed to differences in the composition of the solutions. Firstly, simulated saliva contained α–amylase and mucin, which were absent in simulated lung fluid. The hydrophobic regions of these organic compounds could interact with the hydrophobic PAHs, phenanthrene, and pyrene, potentially reducing the levels of these sorbates in the liquid phase. Secondly, the simulated lung fluid has a higher ionic strength, which could lead to the binding of cations with functional groups of humic acid. This interaction would obstruct the hydrophobic sorption regions, reducing the distribution coefficient and the amount of adsorbed PAHs [27]. A similar result was observed by Luo et al. [28], who proposed that this effect was due to the aggregation of humic acid. As ionic strength increased, the hydrophobic interactions between humic acid molecules were enhanced, potentially leading to the formation of a colloidal structure with a hydrophobic core, causing humic acid to flocculate or precipitate and, thereby, limiting the accessibility of sorbate molecules. Thirdly, the content of dissolved organic matter (DOM) could vary with elevated ionic strength, as the addition of cations would induce the formation of micelle-like structures of DOM [29]. The intra- and/or interaction of DOM with soil particles may block certain sorption sites, leading to lower solid concentration levels of sorbates. Moreover, DOM, as a heterogeneous organic substance, can provide active adsorption sites for PAHs and may even exhibit a stronger affinity for organic compounds than the soil itself [30,31]. This preferential partitioning of PAHs into DOM could result in relatively higher aqueous concentrations of PAHs.

### 3.5. Impact of freeze-thaw Cycle

The freeze-thaw cycles had a pronounced effect on PAH bioaccessibility (*p* < 0.01), confirming our initial hypothesis. The group without freeze-thaw treatment (FT0) exhibited significantly higher release rates, ranging from 39.89% to 74.46% (Table 5 and Table 6). In contrast, groups subjected to 15 (FT15) and 30 (FT30) freeze-thaw cycles showed a general decrease in release rates with increasing cycle frequency (Figure 3).

The bioaccessibility range for FT15 was 32.40% to 51.44%, and for FT30, it was 28.99% to 51.58%. The adsorption reversibility of PAHs decreased with the increasing frequency of freeze-thaw cycles (*p* < 0.05). The group without freeze-thaw treatment (FT0) had the highest reversible adsorbing fraction, with values ranging from 27.60% to 62.29%. In contrast, the group subjected to 15 freeze-thaw cycles (FT15) presented a lower reversible adsorbing fraction, ranging from 19.28% to 50.60%. The group with 30 freeze-thaw cycles (FT30) exhibited the lowest reversible adsorbing fraction, with a range of 18.50% to 43.12% (Table 3). This trend indicates that the freeze-thaw cycles could induce the aging effect in hydrophobic organic compounds, potentially reducing their environmental health risks.

The bioavailability and toxicity of HOCs in soil or sediment gradually decrease over time, a process referred to as “aging” or “sequestration.” The underlying mechanisms can be broadly categorized into two processes. Firstly, HOCs rapidly adsorb onto the surfaces of soil particles and then slowly distribute into the interior of the solid organic matter, complicating their desorption and bioaccessibility. Secondly, HOC molecules “fall into” the micropores of soil particles and enter deeper adsorption sites over time, resulting in the sequestration of HOCs in the soil matrix. Additionally, the formation of strong covalent or hydrogen bonds between the organic compounds and soil humus is also believed to contribute to the aging effect [32,33,34,35,36].

In the current study, the aging phenomenon was observed to impact the bioaccessibilities of phenanthrene and pyrene, with notable declines attributed to freeze-thaw cycles. These cycles could alter the physical structure of the soils, characterized by a reduction in soil bulk density, penetration resistance, and an increase in soil looseness [37,38]. These structural changes affect soil properties, particularly the micropore structure, making it more difficult for HOC molecules to escape, even under conditions of elevated solution temperature and extended contact duration. Furthermore, following freeze-thaw cycles, the long-chain structure of the dissolved fraction of DOM can be disrupted and transformed into a more condensed form of carbon, referred to as “hard carbon.” This transformation results in increased aromaticity and significant enhancement of the adsorption capacity and irreversibility of HOCs. This enhancement could be evidenced by the observed decrease in rapid desorbing rates, indicating that the fate of PAHs in soils is influenced by freeze-thaw cycles. It is worth noting that no linear relationship was found between the decrease in bioaccessibility and the frequency of freeze-thaw cycles, suggesting that the impact of these cycles on the bioaccessibilities of PAHs might be limited. Further research involving more frequent and longer-duration freeze-thaw cycles is necessary to validate this hypothesis.

### 3.6. Implications of PAHs Desorption Kinetics and Aging on Health Risks

The rapid desorption phase of PAHs, governed by the limits of diffusion, plays a critical part in biodegradation and toxicological reactions [33,39]. The kinetic curves and parameters derived from the present study suggest that an incubation period of 24 h could be generally sufficient to evaluate the potential human health risks associated with PAHs. However, the aging effect on the bioaccessibilities of PAHs is complex and covaries with the properties of the sorbates, sorbents, and solutions involved. Research focusing on the re-release of PAHs from environmental media within the human body is crucial for an accurate assessment of the health risks posed by these pollutants. Utilizing the total concentration of pollutants in environmental media to calculate bioaccessibility would result in an overestimation of the associated risks. Furthermore, in the natural environment, soil and other environmental media are subject to epigenetic geochemical processes, which could alter their physical and chemical properties. Consequently, the fate, the environmental risk, and the human health risk of PAHs can change accordingly. A precise understanding of the desorption mechanisms of PAHs and the factors influencing the two-phasic desorption process is of paramount importance for studying the trends and risks of these pollutants in the environment. Such knowledge can inform the development of effective land restoration plans and land management strategies.

## 4. Conclusions

An in vitro methodology was employed to assess the impact of freeze-thaw cycles on the bioaccessibilities of particle-bound PAHs. This study reveals that both the releasing portions and the fractions representing potential human health risks decreased in a nonlinear manner with increasing freeze-thaw cycles. The re-release processes of PAHs from soil particles were influenced by the properties of the PAHs, the nature of the soil, and the composition of the simulated body fluids. PAHs with a higher number of benzene rings demonstrated a stronger affinity for soil particles, attributable to their increased hydrophobicity and larger molecular weight, resulting in relatively lower bioaccessibility levels. The characteristics of SOM were another constraint on the processes. Soil with a higher content of “hard carbon” demonstrated greater binding capacity with PAH molecules and exhibited enhanced stability against environmental perturbations. Furthermore, the findings imply that PAHs may pose a higher health risk in the respiratory tract compared to the oral cavity.

## Figures and Tables

**Table 1:** Composition and properties of simulated body fluids studied [13,14].

Composition	Lung Fluid (mg L^−1^)	Saliva (mg L^−1^)
magnesium chloride	95	–
sodium chloride	6019	290
potassium chloride	298	895
disodium hydrogen phosphate	126	–
sodium sulphate	63	570
calcium chloride dihydrate	368	–
sodium acetate	574	–
sodium hydrogen carbonate	2604	–
sodium citrate dihydrate	97	–
potassium thiocyanate	–	200
sodium dihydrogen phosphate	–	885
urea	–	200
uric acid	–	15
α–amylase	–	145
mucin	–	5
pH	7.2 ± 0.1	6.4 ± 0.1

**Table 2:** Fitted parameters of the two–fraction model of phenanthrene.

Compound	Group	F_rap_ (%)		k_rap_ (h^−1^)		k_slow_ (10^−4^ h^−1^)	
		FS	CS	FS	CS	FS	CS
phenanthrene	simulated lung fluid–FT0	57.90	50.24	1.23	0.73	12.60	13.10
	simulated lung fluid–FT15	42.91	39.07	3.44	1.91	3.47	4.26
	simulated lung fluid–FT30	40.13	37.06	2.56	2.23	1.48	7.38
	simulated saliva–FT0	62.29	47.72	1.20	0.99	6.03	16.10
	simulated saliva–FT15	50.60	35.50	4.97	2.07	2.24	3.79
	simulated saliva–FT30	43.12	34.00	4.06	2.49	2.45	2.48

**Table 3:** Fitted parameters of the two–fraction model of pyrene.

Compound	Group	F_rap_ (%)		k_rap_ (h^−1^)		k_slow_ (10^−4^ h^−1^)	
		FS	CS	FS	CS	FS	CS
pyrene	simulated lung fluid–FT0	41.58	32.87	0.93	5.79	4.20	1.90
	simulated lung fluid–FT15	31.31	24.80	1.73	0.37	5.14	3.37
	simulated lung fluid–FT30	30.38	23.00	0.85	0.83	3.16	2.81
	simulated saliva–FT0	39.00	27.60	0.68	6.90	3.99	2.08
	simulated saliva–FT15	27.11	19.28	1.43	0.66	1.73	2.22
	simulated saliva–FT30	26.43	18.50	0.93	1.12	1.23	1.56

Notes: FS—ferrallitic soil; CS—calcareous soil; FT—freeze-thaw cycle; F_rap_—the rapid desorbing fractions; k_rap_—the rate constants of rapid desorption compartment; k_slow_—the rate constants of slow desorption compartment.

**Table 4:** Selected physicochemical properties of the two target soils [19].

Soil	Organic Fractions			Organic Constituents			Particle Size		
	FA	HA	Humin	TOC	C/N *	H/C *	Sand	Silt	Clay
	(g kg^−1^)	(g kg^−1^)	(g kg^−1^)	(wt%)			(%)	(%)	(%)
FS	2.30	7.90	11.47	2.01	15.02	9.11	13.4	17.7	68.9
CS	5.17	6.70	14.97	3.12	10.81	4.00	24.8	52.4	22.8

Notes: *—Atomic ratio; FS—ferrallitic soil; CS—calcareous soil; FA—fulvic acid; HA—humic acid; TOC—total organic carbon.

**Table 5:** Bioaccessibilities of phenanthrene across all scenarios.

Compound	Soil	Simulated Body Fluid	freeze-thaw Cycle	Bioaccessibility Range
phenanthrene	FS	lungf fluid	0	25.41–75.46%
			15	30.17–51.44%
			30	25.01–48.62%
		saliva	0	24.23–59.50%
			15	31.25–46.49%
			30	26.10–44.69%
	CS	lungf fluid	0	18.82–72.58%
			15	19.05–51.58%
			30	19.16–47.47%
		saliva	0	20.86–62.41%
			15	19.17–46.75%
			30	19.45–42.03%

**Table 6:** Bioaccessibilities of pyrene across all scenarios.

Compound	Soil	Simulated Body Fluid	freeze-thaw Cycle	Bioaccessibility Range
pyrene	FS	lungf fluid	0	22.73–54.37%
			15	17.48–50.30%
			30	11.98–46.47%
		saliva	0	17.46–52.42%
			15	13.33–47.65%
			30	8.87–43.66%
	CS	lungf fluid	0	11.80–47.23%
			15	5.61–46.30%
			30	6.87–42.48%
		saliva	0	8.55–38.89%
			15	6.87–32.40%
			30	5.02–28.99%

## Data Availability

The data presented in this study are available on request from the corresponding author.
